# Postoperative Patient-Reported Visual Symptoms After Robot-Assisted Laparoscopic Radical Prostatectomy in Steep Trendelenburg: A Prospective Single-Center Observational Cohort Study

**DOI:** 10.3390/life16050704

**Published:** 2026-04-22

**Authors:** Iacopo Cappellini, Francesca Tabani, Laura Campiglia, Elena Schirru, Vittorio Pavoni

**Affiliations:** Department of Emergency and Critical Care Medicine, Ospedale Santo Stefano, USL Toscana Centro, 59100 Prato, Italy; francesca.tabani@uslcentro.toscana.it (F.T.); laura.campiglia@uslcentro.toscana.it (L.C.); elena.schirru@uslcentro.toscana.it (E.S.); vittorio.pavoni@uslcentro.toscana.it (V.P.)

**Keywords:** robot-assisted laparoscopic radical prostatectomy, perioperative visual loss, steep Trendelenburg, optic nerve sheath diameter, intraocular pressure, patient-reported outcomes

## Abstract

Background: Robot-assisted laparoscopic radical prostatectomy (RALP) requires prolonged steep Trendelenburg positioning, which increases intraocular and intracranial pressure. Although transient visual field defects have been documented after RALP using objective perimetric testing, data on patient-reported visual outcomes remain limited. We hypothesized that intraoperative optic nerve sheath diameter (ONSD) measurements and hemodynamic variables would be associated with postoperative patient-reported visual symptoms. Methods: This prospective, single-center observational cohort study enrolled consecutive adult patients undergoing RALP between March and September 2023 at Ospedale Santo Stefano, Prato, Italy. Patients with pre-existing glaucoma, ocular disease, or intracranial hypertension were excluded. Intraoperative ONSD was measured by transorbital ultrasound at three time points: before Trendelenburg (t1), 30 min after Trendelenburg (t2), and at end of Trendelenburg (t3). Postoperative visual symptoms were assessed at ≥1 month follow-up using the validated Catquest-9SF questionnaire. Rasch analysis converted ordinal responses to interval-level measures. Logistic regression explored associations between visual complaints and intraoperative predictors (Rasch scores, lowest mean arterial pressure [MAP], maximum ONSD). Results: Fifty-five patients were enrolled. Six patients (10.9%) reported new subjective visual symptoms at follow-up. Rasch-transformed scores were associated with the presence of these symptoms (coefficient 1.38; *p* < 0.05). Lowest intraoperative MAP (*p* = 0.081) and maximum ONSD (*p* = 0.811) did not reach statistical significance as independent factors. Conclusions: Patient-reported visual symptoms occurred in approximately 11% of patients after RALP. Postoperative Rasch-transformed visual function scores correlated with these complaints. While intraoperative ONSD was not associated with visual outcomes, the potential role of intraoperative hypotension requires further investigation in larger, powered cohorts.

## 1. Introduction

The widespread adoption of robotic surgery has introduced new perioperative challenges for anesthesiologists. Robotic procedures are often longer than open surgery, and prolonged steep Trendelenburg positioning may cause cephalic venous congestion, facial edema, and increased intraocular pressure (IOP), resembling the physiological derangements described in prolonged prone spine surgery [1,2]. Concerns have therefore emerged regarding a possible association between robotic/laparoscopic surgery in steep Trendelenburg and perioperative visual loss (POVL), including ischemic optic neuropathy (ION). Although only a limited number of POVL cases have been reported in this setting, this may relate to historically shorter operative durations and lower blood loss compared with high-risk procedures such as complex spine surgery; as robotic techniques expand to longer and more complex operations, the burden of visual complications may increase [3].

Robot-assisted laparoscopic radical prostatectomy (RALP) is among the most commonly performed robotic procedures worldwide. While it offers advantages such as reduced postoperative pain and blood loss, the steep Trendelenburg position (typically ≥30°) required for pelvic exposure can significantly increase IOP, potentially lowering ocular perfusion pressure and predisposing to ocular complications [4,5,6,7].

## 2. Background

### 2.1. Perioperative Visual Loss: Definition and Epidemiology

Perioperative visual loss (POVL) refers to permanent visual impairment or complete vision loss temporally associated with surgery under general anesthesia, occurring within the perioperative period from immediate preoperative assessment through discharge from acute care [8]. POVL is feared by patients and clinicians because common etiologies lack effective treatment and are often associated with poor visual recovery, with a devastating impact on functional status and quality of life [1,9].

The study of POVL remains limited by its low incidence, lack of suitable animal models, and the absence of reliable intraoperative monitoring of the visual pathways under anesthesia [1,9]. The incidence of POVL after non-ocular surgeries ranges from 0.002% of all surgeries to as high as 0.2% of cardiac and spine surgeries [1]. In a large retrospective study of more than 5.6 million patients in the United States, cardiac surgery had the highest rate of POVL (8.64/10,000), followed by spinal fusion (3.09/10,000) [10]. In cardiac surgery specifically, the incidence of ischemic optic neuropathy was 0.014% (1.43 per 10,000 procedures) [11].

### 2.2. Mechanisms and Etiologies of POVL

The most commonly reported ocular injury during anesthesia is corneal abrasion and/or exposure keratopathy, which rarely results in permanent visual loss [1,8]. By contrast, the leading causes of permanent POVL include anterior and posterior ischemic optic neuropathy (AION, PION), central retinal artery occlusion (CRAO) and other retinal vascular occlusions, and cortical blindness [1,8,12]. In non-ocular surgery under general anesthesia, the optic nerves represent the most frequent site of permanent injury, and ischemia is considered the predominant mechanism [9,12].

Ischemic optic neuropathy is now the most frequently reported diagnosis associated with permanent POVL, most commonly after prone spine surgery [13,14]. AION typically presents with acute optic disc edema, hypoperfusion, and frequent peripapillary hemorrhages on early funduscopic examination, whereas PION usually shows an initially normal fundus; in both conditions, pupillary reactivity is abnormal [15]. In cardiac surgery, AION appears more frequent than PION, whereas PION predominates after prolonged prone spine procedures and bilateral radical neck dissection [12]. PION is also associated with procedures that increase venous pressure in the head, including prone and Trendelenburg positioning [1,2].

Retinal vascular occlusions (CRAO/branch retinal vein occlusion) are more commonly associated with procedures at risk of embolization near facial vessels or direct globe compression, classically in prone positioning [1,8]. Cortical blindness results from retrochiasmal injuries causing homonymous hemianopia (unilateral lesions) or cortical visual impairment (bilateral lesions) with preserved pupillary light reflexes; these injuries are usually ischemic, most often embolic infarctions in the posterior cerebral circulation, and may also occur in severe hypotension or high embolic-risk settings such as cardiac surgery [1].

### 2.3. Pathophysiology and Risk Factors

Perioperative ION has been reported after a broad range of non-ocular surgeries, including spine surgery, cardiac surgery, radical neck dissection, and major vascular, abdominal, and orthopedic procedures [1,9]. Despite intuitive assumptions, traditional vascular risk factors (hypertension, diabetes, hyperlipidemia) do not consistently discriminate cases from controls; notably, ION may occur in relatively healthy individuals [14]. As a result, multiple intraoperative factors have been proposed as causal or contributory, including hypotension (absolute or relative), anemia, blood loss, hypoxemia, hemodilution, hypovolemia, large crystalloid volumes, vasopressor use, head position relative to the heart, elevated venous pressure, increased IOP, direct globe compression, and individual susceptibility [1,2,13,14].

Attributing causality remains difficult. Many candidate factors are common during surgery yet POVL is rare, implying that combinations of factors and patient susceptibility likely interact [9,12]. Moreover, some findings (e.g., periorbital edema after prone procedures) may be epiphenomena rather than direct mechanisms. Preventive strategies are therefore challenging, particularly because broad hemodynamic “tightening” for all patients could induce harm, and vasopressor-driven increases in systemic pressure might theoretically worsen microvascular perfusion in susceptible beds.

### 2.4. Practice Advisory for POVL Prevention

The American Society of Anesthesiologists (ASA) Task Force on Perioperative Visual Loss, in collaboration with the North American Neuro-Ophthalmology Society and the Society for Neuroscience in Anesthesiology and Critical Care, published an updated Practice Advisory in 2019 for POVL associated with spine surgery [8]. Key recommendations include individualized preoperative risk assessment (including anemia and vascular risk factors), selective patient counseling regarding POVL risk, careful intraoperative blood pressure management, monitoring of hemoglobin/hematocrit when clinically indicated, judicious fluid and transfusion strategies, meticulous patient positioning to avoid direct ocular pressure, and consideration of staged procedures for high-risk patients requiring prolonged surgery [8]. Although these advisories are not standards or absolute requirements, they summarize the available literature and expert consensus in a field where randomized evidence is limited by the rarity of the outcome.

### 2.5. Physiological Effects of Steep Trendelenburg Positioning During RALP

#### 2.5.1. Cardiovascular and Respiratory Effects

The combination of pneumoperitoneum and steep Trendelenburg positioning required for RALP induces significant cardiovascular, respiratory, and neurologic effects [16,17]. Cardiovascular changes relate to increased intra-abdominal pressure and potential vena caval compression, with variable effects on venous return and cardiac output; Trendelenburg positioning may augment venous return from the lower extremities, modifying preload and arterial pressure [17,18]. Kalmar et al. demonstrated that steep Trendelenburg combined with CO_2_ pneumoperitoneum significantly affects cardiovascular, cerebrovascular, and respiratory homeostasis during robotic prostatectomy [17].

Respiratory effects include diaphragmatic displacement and reduced functional residual capacity with increased atelectasis risk, particularly in patients with baseline lung disease or obesity [19,20,21,22]. Tharp et al. showed that body habitus and dynamic surgical conditions independently impair pulmonary mechanics during robotic-assisted laparoscopic surgery [19]. Aspiration risk may also be increased due to gastroesophageal reflux in the head-down position.

#### 2.5.2. Effects on Intracranial and Intraocular Pressure

Steep Trendelenburg positioning may increase intracranial pressure (ICP) via increased cerebral venous pressure and impaired venous outflow, increasing intracranial blood volume [16,17]. These changes can theoretically reduce cerebral perfusion pressure, increase risk of ischemic injury, and in vulnerable patients increase risk of intracranial hemorrhage [16,23].

Multiple studies have documented significant IOP elevation during RALP in steep Trendelenburg. Hoshikawa et al. reported time-dependent IOP increases, reaching up to 36 mmHg during prolonged procedures [6]. Awad et al. found IOP increases up to approximately 30 mmHg, although no permanent structural or functional ocular changes were detected at 3-month follow-up [5]. Raz et al. demonstrated that a modified Z-Trendelenburg position could attenuate IOP elevation compared with standard steep Trendelenburg [4].

The mechanism of IOP elevation during steep Trendelenburg is multifactorial and includes increased episcleral venous pressure secondary to cephalic venous congestion, choroidal engorgement, and possibly increased aqueous humor production [23,24]. The combination of elevated IOP and potential reductions in mean arterial pressure may compromise ocular perfusion pressure (OPP = MAP − IOP), theoretically predisposing to ischemic injury of the optic nerve or retina [16,23].

#### 2.5.3. Optic Nerve Sheath Diameter as a Surrogate of Intracranial Pressure

Given the potential for ICP alterations during steep Trendelenburg and the need for non-invasive monitoring tools suitable for the operating room, transorbital ultrasound measurement of optic nerve sheath diameter (ONSD) has gained attention as a bedside technique [25,26]. The optic nerve sheath is contiguous with the subarachnoid space; when ICP rises, cerebrospinal fluid pressure can distend the sheath, increasing ONSD [25,27].

Systematic reviews and meta-analyses have demonstrated that ONSD ultrasonography has high diagnostic accuracy for detecting increased ICP, with pooled sensitivity of 90–97% and specificity of 86–87% [25,26]. In many clinical protocols, ONSD values above approximately 5.0–5.6 mm are considered suggestive of raised ICP, although optimal cut-off values ranging from 5.6 to 6.3 mm have been associated with higher specificity without compromising sensitivity [26,28,29]. The technique requires standardization and contextual clinical interpretation [25,27].

Several studies have examined ONSD changes during RALP. Kim et al. found that propofol-based anesthesia was associated with smaller ONSD increases compared with sevoflurane during robotic surgery in steep Trendelenburg [30]. De Bernardo et al. reviewed optic nerve changes detected with ocular ultrasonography during various surgical procedures, noting the utility of this technique for assessing ICP changes intraoperatively [31].

## 3. Materials and Methods

### 3.1. Study Design and Population

This was a prospective, single-center, no-profit observational cohort study conducted at the Department of Anesthesia and Intensive Care (SOC Anestesia e Rianimazione), Ospedale Santo Stefano, Prato, Italy. Consecutive adult patients undergoing RALP between March and September 2025 were screened for eligibility. This study was conducted and reported in accordance with the Strengthening the Reporting of Observational Studies in Epidemiology (STROBE) guidelines for cohort studies.

Inclusion criteria:

- Written informed consent;
- Age > 18 years;
- Negative history for glaucoma and/or other ocular surgical disease;
- Negative history for intracranial hypertension.

Exclusion criteria:

- Refusal or inability to provide informed consent;
- Age < 18 years;
- History of glaucoma or other significant ocular disease;
- History of intracranial hypertension.

The study was conducted in accordance with the Declaration of Helsinki and approved by the local ethics committee. All patients provided written informed consent prior to enrollment.

### 3.2. Perioperative Anesthetic Management

All patients underwent general anesthesia, with addition of a locoregional technique (intrathecal morphine) when feasible to optimize postoperative analgesia. Anesthetic induction included fentanyl 1–2 µg/kg or remifentanil 0.05 µg/kg/min, propofol 1–2 mg/kg, and rocuronium 0.6–1 mg/kg. Maintenance was provided with a volatile anesthetic (sevoflurane or desflurane) titrated according to anesthetic depth assessed by processed EEG monitoring (BIS or PSI, target 40–60).

Antibiotic prophylaxis was administered with a first-generation cephalosporin; clindamycin was used in case of suspected or confirmed penicillin allergy. Postoperative nausea and vomiting (PONV) prophylaxis was provided according to institutional protocol. After induction, an arterial catheter was placed for continuous invasive blood pressure monitoring and serial arterial blood gas analyses (including hemoglobin trend, lactate, and electrolytes).

When locoregional analgesia was used, intrathecal morphine (0.2 mg diluted in 3 mL normal saline) was administered before induction at the L1–L2, L2–L3, or L3–L4 interspace using a 25 G or 27 G spinal needle.

### 3.3. Surgical Positioning

After induction of anesthesia and placement of monitoring, patients were positioned supine on the operating table with arms tucked at the sides. Pneumoperitoneum was established with CO_2_ insufflation to a target pressure of 12–15 mmHg. Steep Trendelenburg positioning (approximately 30–35°) was then initiated for pelvic exposure. Eye protection was ensured with tape closure of the eyelids; no additional eye shields or goggles were used routinely.

### 3.4. Transorbital Ultrasound Protocol

Transorbital ultrasound (TOS) was performed intraoperatively at predefined time points to evaluate optic nerve metrics. Images were acquired using a high-frequency linear probe (7.5–12 MHz) in B-mode, placed gently over the closed upper eyelid with a layer of ultrasound gel. Care was taken to apply minimal pressure to avoid iatrogenic IOP elevation.

Sonographically, the optic nerve is identified as a hypoechoic linear structure posterior to the globe; the surrounding subarachnoid space appears anechoic/hypoechoic and is bordered by a hyperechoic rim corresponding to dura mater and periorbital fat. ONSD measurements were performed at 3 mm posterior to the optic disc, measuring the transverse diameter of the optic nerve sheath perpendicular to the nerve axis. Bilateral measurements were obtained and averaged.

TOS was performed at three predefined time points:

- t1: Immediately after induction, before Trendelenburg positioning (baseline);
- t2: 30 min after initiation of steep Trendelenburg positioning;
- t3: At the end of steep Trendelenburg positioning, before returning to supine position.

An ONSD > 5 mm was considered suggestive of elevated ICP, consistent with published thresholds [25,26].

### 3.5. Data Collection

Data were collected anonymously by the principal investigator using a standardized electronic case report form and the anesthesia chart. The following variables were recorded:

Demographic and baseline characteristics included age, BMI, and ASA physical status (reported as categorical distribution).

Intraoperative variables: anesthesia start and end time, total anesthesia duration, duration of steep Trendelenburg positioning, mean processed EEG value (BIS/PSI), lowest intraoperative mean arterial pressure, lowest intraoperative hemoglobin, estimated blood loss, total crystalloid volume administered, vasopressor use, ONSD measurements at t1, t2, and t3, and use of intrathecal morphine.

### 3.6. Postoperative Assessment

All enrolled patients underwent a structured postoperative telephone interview at least 1 month after surgery. The interview included administration of the Catquest-9SF questionnaire (Italian validated version) to capture subjective difficulties related to vision in daily life [32,33,34].

The Catquest-9SF is a validated, Rasch-calibrated patient-reported outcome measure originally developed for cataract surgery outcomes assessment but applicable to broader visual function evaluation. It consists of 9 items assessing perceived difficulty with vision-dependent activities and global visual satisfaction. Response options are ordinal (e.g., “no difficulty,” “some difficulty,” “great difficulty,” “cannot do because of vision”). The questionnaire has demonstrated good psychometric properties including ordered response thresholds, adequate precision, and unidimensionality across multiple populations [32,33,34,35,36,37].

Patients were specifically asked about any new visual symptoms experienced since surgery, including blurred vision, visual field defects or blind spots, difficulty with color perception, difficulty with night vision, and any other visual complaints.

### 3.7. Statistical Analysis

Rasch measurement theory converted ordinal Catquest-9SF responses into interval-level measures (theta). In this study, higher theta values represent greater perceived visual difficulty (worse visual function) to align with the logistic regression model. A logistic regression model explored associations between postoperative visual complaints and three variables: Rasch person measures, lowest intraoperative MAP, and maximum ONSD. Results are reported as regression coefficients (β), odds ratios (OR) with 95% confidence intervals (CI), and *p*-values.

A logistic regression model was fitted to explore associations between postoperative visual complaints (binary dependent variable: yes/no) and three intraoperative predictors: Rasch score (person measure), lowest intraoperative MAP, and maximum ONSD during surgery. Results are reported as regression coefficients (β), odds ratios (OR) with 95% confidence intervals (CI), and *p*-values. Statistical significance was defined as *p* < 0.05 (two-tailed). All analyses were performed using R statistical software (version 4.3.0).

### 3.8. Ethical Approval

This study was approved by the Comitato Etico Regionale per la Sperimentazione Clinica della Toscana—Sezione Area Vasta Centro (Tuscany Regional Ethics Committee—Area Vasta Centro Section) as Protocol ID: VA-RALP (n. 25336/oss) on 2 July 2024. Chairman: Prof. Matteo Galletti.

## 4. Results

### 4.1. Patient Characteristics

A total of 55 consecutive patients undergoing RALP were enrolled during the study period (March–September 2023). No patients were excluded after enrollment, and all completed the follow-up assessment. Baseline demographic and clinical characteristics are presented in Table 1.

### 4.2. Intraoperative Variables

Intraoperative and surgical variables are summarized in Table 2.

### 4.3. Optic Nerve Sheath Diameter Measurements

ONSD measurements were successfully obtained at all three time points in all 55 patients. Results are presented in Table 3.

ONSD increased progressively from baseline (t1) through the end of Trendelenburg positioning (t3), consistent with expected physiological changes during steep head-down positioning.

### 4.4. Incidence of Postoperative Visual Symptoms

This study found that 10.9% of patients reported new subjective visual symptoms post-RALP. Rasch-transformed scores were significantly associated with these complaints, acting as a concurrent measure of postoperative visual satisfaction.

### 4.5. Rasch Analysis of Catquest-9SF Responses

Rasch analysis was performed using selected items from the Catquest-9SF questionnaire relevant to this perioperative context. These items captured key domains of patient-perceived visual functioning, including color perception, night vision, and clarity of vision.

The Rasch model enabled estimation of person measures (theta) reflecting the latent trait of visual ability/visual satisfaction on an interval scale. The distribution of theta values indicated meaningful heterogeneity in postoperative visual function within the cohort, with higher theta values representing better visual ability and lower values representing greater difficulty.

**Figure 1:**
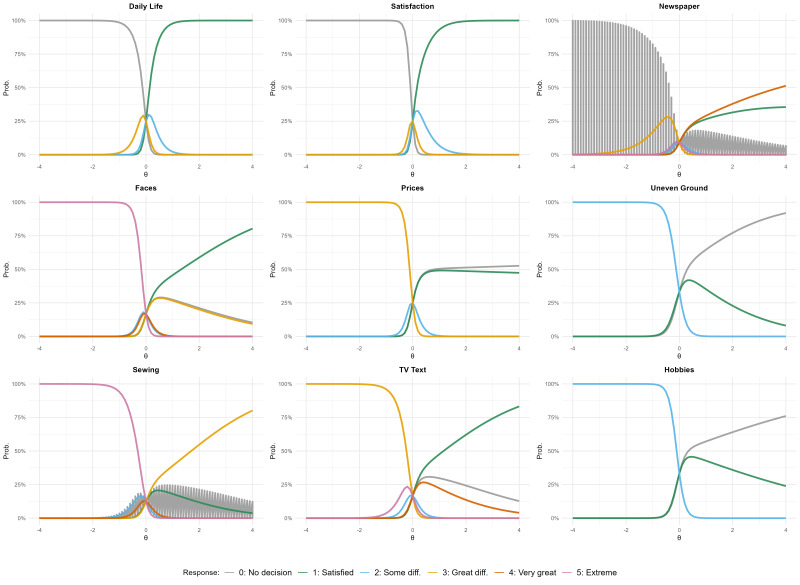
Item characteristic curves (ICC). Curves peak at different θ values, showing item difficulty. Categories with peaks at higher θ (right) are easier items (requiring better visual ability to endorse lower-difficulty responses).

Item characteristic curves (ICCs) were examined to characterize the response behavior of questionnaire items. Categories with a maximum probability approaching 1 at positive theta values indicated that individuals with better perceived vision tended to select that category consistently. Conversely, categories whose peak probability remained below 1 (e.g., ~0.75) despite positive theta values suggested more heterogeneous response patterns, indicating that even individuals with relatively good perceived vision may distribute responses across adjacent categories for certain items (Figure 1).

### 4.6. Logistic Regression Analysis

A logistic regression model was fitted to explore associations between postoperative visual complaints (dependent variable) and three intraoperative predictors. Results are presented in Table 4.

**Figure 2:**
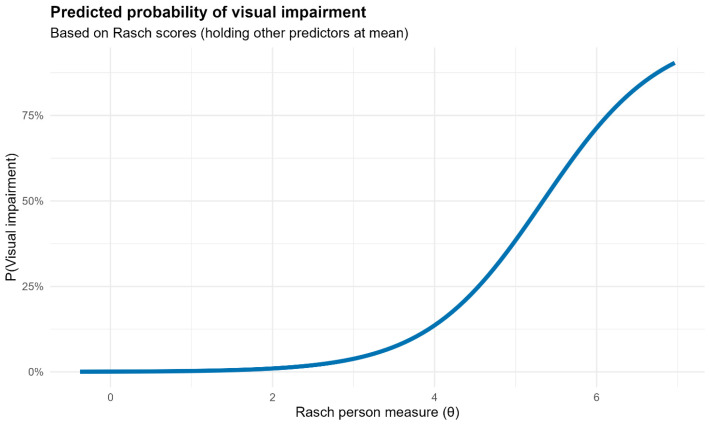
Logistic regression for visual impairment.

**Rasch score** demonstrated a statistically significant association with the likelihood of reporting postoperative visual symptoms. Specifically, for each one-point increase in Rasch score (indicating worse visual function), the log-odds of reporting postoperative visual alterations increased by approximately 1.38 (Figure 2).

In our model, the lowest intraoperative MAP did not reach the threshold for statistical significance (*p* = 0.081). While this prevents definitive clinical conclusions, the finding aligns with the physiological hypothesis that reduced arterial pressure, combined with elevated IOP, might compromise ocular perfusion. However, given the low event rate (n = 6), this result should be considered hypothesis-generating rather than confirmatory.

The lack of association between maximum ONSD and visual symptoms (*p* = 0.811) suggests that transient optic nerve sheath distension alone is not a reliable indicator of patient-perceived visual changes. ONSD reflects intracranial pressure changes, which may not directly correlate with the multifactorial nature of patient-reported visual satisfaction.

## 5. Discussion

### 5.1. Principal Findings

This prospective observational study found that 10.9% of patients (6/55) reported new subjective visual difficulties at least 1 month after undergoing RALP performed in steep Trendelenburg positioning. Rasch-transformed Catquest-9SF scores were significantly associated with postoperative visual complaints, whereas maximum intraoperative ONSD was not a significant predictor. Lowest intraoperative MAP showed a trend toward significance, suggesting a potential role for intraoperative hypotension in the development of visual symptoms.

### 5.2. Comparison with Existing Literature

The incidence of patient-reported visual symptoms in our cohort (10.9%) is lower than the rates of objective visual field defects reported in previous studies using perimetric testing. Taketani et al., in a prospective study of 25 patients (50 eyes) undergoing RALP, found transient visual field defects in 28% of patients using Humphrey visual field 30-2 testing; all defects resolved within 3 months [7]. Similarly, Kakutani et al. reported transient postoperative visual field defects in 17.3% of 98 patients (24 eyes of 17 patients), including patients with pre-existing ocular diseases; most defects recovered within 3 months [38].

The discrepancy between our findings and these studies may be explained by methodological differences. Our study assessed patient-reported symptoms using a validated questionnaire (Catquest-9SF), whereas previous studies employed objective perimetric testing capable of detecting subclinical defects that patients may not perceive or report. This suggests that while objective visual field abnormalities may be relatively common after RALP, only a subset of these translate into patient-perceived visual difficulties.

Importantly, several studies have demonstrated that despite significant intraoperative IOP elevation during steep Trendelenburg positioning, no permanent structural or functional ocular changes were detected at long-term follow-up. Awad et al. found no significant differences in retinal nerve fiber layer thickness (RNFLT), ganglion cell complex (GCC) thickness, or visual field parameters at 3 months postoperatively, despite IOP increases of up to approximately 30 mmHg during surgery [5]. Mizumoto et al. reported no significant disorders in ocular structural and functional parameters at 3 and 6 months after RALP [39]. Hoshikawa et al. confirmed that while IOP increased in a time-dependent fashion during steep Trendelenburg (reaching up to 36 mmHg), visual function showed no significant change postoperatively in patients without pre-existing ocular disease (Table 5) [6].

**Table 5:** Comparison with published literature on visual outcomes after RALP.

Study	Year	N	Visual Assessment	Incidence of Visual Changes	Long-Term Outcome
Hoshikawa et al. [6]	2014	31 eyes	VA, RNFL	No significant change	No complications at 1 month
Taketani et al. [7]	2015	50 eyes	Humphrey VF 30-2	28% transient VF defects	Recovery within 3 months
Mizumoto et al. [39]	2017	44 eyes	Humphrey VF, OCT	No significant change	No change at 3–6 months
Awad et al. [5]	2020	52 pts	VA, RNFL, GCC	No significant change	No change at 3 months
Kakutani et al. [38]	2020	98 pts	Humphrey VF	17.3% transient VF defects	Recovery within 3 months
Present study	2023	55 pts	Catquest-9SF (PRO)	10.9% patient-reported	—

Abbreviations: GCC = ganglion cell complex; OCT = optical coherence tomography; PRO = patient-reported outcome; pts = patients; RNFL = retinal nerve fiber layer; VA = visual acuity; VF = visual field.

### 5.3. Pathophysiological Considerations

The steep Trendelenburg position combined with pneumoperitoneum induces significant physiological changes that may affect ocular perfusion. IOP increases progressively during RALP, with values reaching 21–30 mmHg at the end of prolonged procedures [6,7]. This IOP elevation is attributed to increased episcleral venous pressure secondary to cephalic venous congestion, choroidal engorgement, and possibly increased aqueous humor production [23,24]. The combination of elevated IOP and potential reductions in mean arterial pressure may compromise ocular perfusion pressure (OPP = MAP − IOP), theoretically predisposing to ischemic injury of the optic nerve or retina [16,23].

Weber et al. reported the first cases of posterior ischemic optic neuropathy (PION) after minimally invasive prostatectomy, including one robotic-assisted case, highlighting that prolonged steep Trendelenburg positioning may increase the risk of this devastating complication [3]. However, permanent POVL after RALP remains exceedingly rare, and the transient visual field defects reported in most studies resolve spontaneously within 1–3 months [7,38].

### 5.4. Role of Intraoperative Hypotension

The trend toward significance observed for (*p* = 0.081) in our logistic regression model is consistent with the pathophysiological rationale that reduced arterial pressure, combined with elevated IOP, may compromise ocular perfusion. The ASA Practice Advisory for POVL associated with spine surgery notes that the literature is equivocal on whether intraoperative hypotension increases the risk of ischemic optic neuropathy, although case reports describe POVL occurring after procedures with hypotensive episodes [8]. Multiple factors have been proposed as risk factors for perioperative ION, including hypotension, anemia, blood loss, prolonged duration in prone/Trendelenburg position, and individual vascular susceptibility [1,14]. Dunker et al. identified intraoperative systemic hypotension as a notable contributing factor in all seven cases of PION in their series [40].

Given the multifactorial nature of POVL and the rarity of the outcome, larger studies with more granular hemodynamic data are needed to clarify the independent contribution of intraoperative hypotension to visual complications after RALP.

### 5.5. ONSD as a Predictor of Visual Outcomes

The absence of a significant association between maximum ONSD and postoperative visual complaints in our cohort suggests that intraoperative optic nerve sheath distension alone may not be sufficient to predict patient-perceived visual difficulties. While ONSD ultrasonography has demonstrated high diagnostic accuracy for detecting increased ICP in neurological settings, with pooled sensitivity of 90–97% and specificity of 86–87%, its predictive value for visual outcomes in the surgical setting remains unclear [25,26].

Several factors may explain this finding. First, ONSD reflects intracranial pressure rather than intraocular pressure or ocular perfusion pressure directly. Second, the relationship between transient ICP elevation and subsequent visual symptoms may be non-linear and influenced by individual susceptibility factors. Third, patient-reported visual symptoms captured by the Catquest-9SF may reflect a broader construct of visual satisfaction that is not fully explained by a single physiological parameter. De Bernardo et al. noted that while ocular ultrasonography is a valuable diagnostic tool for assessing ICP changes during surgical procedures, its use should be coupled with standardized techniques for accurate assessment [31].

### 5.6. Strengths and Limitations

This study has several strengths, including its prospective design, use of a validated patient-reported outcome measure (Catquest-9SF), application of Rasch analysis to convert ordinal responses to interval-level measures, and systematic intraoperative ONSD monitoring at predefined time points.

This study is limited by a small sample size (N = 55), which resulted in a low number of symptomatic events (n = 6). This sparse data limits the statistical power of the logistic regression and increases the risk of overfitting. Furthermore, the absence of a preoperative Catquest-9SF baseline and objective ophthalmic testing (e.g., perimetry or OCT) means that subclinical defects may have been missed, and the “new” nature of the symptoms relies solely on patient recall.

### 5.7. Clinical Implications

Our findings support the importance of individualized postoperative assessment of visual function in patients undergoing RALP in steep Trendelenburg positioning. While permanent POVL after RALP is rare, transient visual symptoms may occur in a meaningful proportion of patients. Preoperative counseling regarding potential visual symptoms, particularly in patients with pre-existing ocular conditions, is recommended [23,38]. Intraoperative strategies to mitigate IOP elevation, such as the modified Z-Trendelenburg position described by Raz et al., periodic return to supine position, and careful blood pressure management, may be considered in high-risk patients or prolonged procedures [4,16,24].

### 5.8. Future Directions

Future research should aim to validate these findings in larger, multicenter cohorts with sufficient power to detect associations between intraoperative variables and visual outcomes. Incorporation of objective visual field testing alongside patient-reported outcomes would provide complementary information on the spectrum of postoperative visual changes. Investigation of modifiable intraoperative factors, including anesthetic technique (propofol vs. sevoflurane), ventilation strategies, and hemodynamic targets, may identify opportunities for risk reduction [30]. Finally, development of risk prediction models incorporating patient-specific factors (age, pre-existing ocular disease, cardiovascular comorbidities) and procedural variables (duration, blood loss, hemodynamic parameters) could support individualized perioperative management.

## 6. Conclusions

This prospective observational study found that patient-reported visual symptoms occurred in approximately 11% of patients after undergoing RALP performed in steep Trendelenburg positioning. Rasch-transformed Catquest-9SF visual function scores were significantly associated with postoperative visual complaints, whereas maximum intraoperative ONSD was not a significant predictor. The lowest intraoperative MAP showed a trend toward significance, suggesting a potential role for intraoperative hypotension. These findings highlight the relevance of individualized postoperative assessment of visual function and warrant validation in larger, multicenter cohorts. Future research should incorporate both objective visual field testing and patient-reported outcomes to comprehensively characterize the spectrum of visual changes after RALP.

## Figures and Tables

**Table 1:** Baseline demographic and clinical characteristics (*n* = 55).

Variable	Value
Age (years), mean ± SD	65.96 ± 8.52
BMI (kg/m^2^), mean ± SD	26.36 ± 3.02
ASA Class II, *n* (%)	50 (90.9%)
ASA Class III, *n* (%)	5 (9.1%)

Abbreviations: ASA = American Society of Anesthesiologists; BMI = body mass index; SD = standard deviation.

**Table 2:** Intraoperative and surgical variables (*n* = 55).

Variable	Value
Surgical duration, min, mean ± SD	174.64 ± 46.88
Mean BIS/PSI value, mean ± SD	50.11 ± 5.34
Lowest intraoperative MAP, mmHg, mean ± SD	58.27 ± 4.09
Lowest intraoperative hemoglobin, g/dL, mean ± SD	12.96 ± 1.35
Estimated blood loss, mL, mean ± SD	267.27 ± 317.14

Abbreviations: BIS = bispectral index; MAP = mean arterial pressure; PSI = patient state index; SD = standard deviation.

**Table 3:** Optic nerve sheath diameter measurements at predefined time points (*n* = 55).

Time Point	Right Eye ONSD, mm, Mean ± SD	Left Eye ONSD, mm, Mean ± SD
t1—Before Trendelenburg (baseline)	4.00 ± 0.67	3.93 ± 0.61
t2—30 min after Trendelenburg	4.29 ± 0.66	4.21 ± 0.65
t3—End of Trendelenburg	4.39 ± 0.61	4.34 ± 0.63
Maximum ONSD during surgery, mean ± SD	4.53 ± 0.65	—

Abbreviations: ONSD = optic nerve sheath diameter; SD = standard deviation.

**Table 4:** Logistic regression analysis: predictors of postoperative visual complaints.

Variable	Coefficient (β)	Odds Ratio (OR)	95% CI	*p*-Value
Rasch score	1.38	3.97	(1.00–15.75)	0.050
Lowest MAP	0.25	1.29	(0.97–1.73)	0.081
Maximum ONSD	0.18	1.20	(0.27–5.32)	0.811

Statistically significant (*p* < 0.05). Abbreviations: MAP = mean arterial pressure; ONSD = optic nerve sheath diameter.

## Data Availability

The data supporting the findings of this study are available from the corresponding author upon reasonable request, subject to ethical approval and patient consent requirements.
